# Do Nonsuicidal Severely Depressed Individuals with Diabetes Profit from Internet-Based Guided Self-Help? Secondary Analyses of a Pragmatic Randomized Trial

**DOI:** 10.1155/2019/2634094

**Published:** 2019-05-14

**Authors:** Sandra Schlicker, Kiona K. Weisel, Claudia Buntrock, Matthias Berking, Stephanie Nobis, Dirk Lehr, Harald Baumeister, Frank J. Snoek, Heleen Riper, David D. Ebert

**Affiliations:** ^1^Friedrich-Alexander University Erlangen-Nürnberg, Germany; ^2^Philipps-University Marburg, Germany; ^3^GET.ON Institute GmbH, Germany; ^4^Leuphana University Lüneburg, Germany; ^5^Ulm University, Germany; ^6^VU University, Amsterdam, Netherlands

## Abstract

**Introduction:**

Diabetes mellitus type 1 and type 2 are linked to higher prevalence and occurrences of depression. Internet-based depression- and diabetes-specific cognitive behavioral therapies (CBT) can be effective in reducing depressive symptom severity and diabetes-related emotional distress. The aim of the study was to test whether disease-specific severity indicators moderate the treatment outcome in a 6-week minimally guided web-based self-help intervention on depression and diabetes (GET.ON Mood Enhancer Diabetes (GET.ON M.E.D.)) and to determine its effectiveness in a nonsuicidal severely depressed subgroup.

**Methods:**

Randomized controlled trial- (RCT-) based data (*N* = 253) comparing GET.ON M.E.D. to an online psychoeducation control group was used to test disease-specific severity indicators as predictors/moderators of a treatment outcome. Changes in depressive symptom severity and treatment response were examined in a nonsuicidal severely depressed subgroup (CES − D > 40; *N* = 40).

**Results:**

Major depressive disorder diagnosis at the baseline (*p*_prf6_ = 0.01), higher levels of depression (Beck Depression Inventory II; *p*_prpo_ = 0.00; *p*_prf6_ = 0.00), and lower HbA_1c_ (*p*_prpo_ = 0.04) predicted changes in depressive symptoms. No severity indicator moderated the treatment outcome. Severely depressed participants in the intervention group showed a significantly greater reduction in depressive symptom severity (*d*_prpo_ = 2.17, 95% Confidence Interval (CI): 1.39-2.96) than the control condition (*d*_prpo_ = 0.92; 95% CI: 0.001-1.83), with a between-group effect size of *d*_prpo_ = 1.05 (95% CI: 0.11-1.98). Treatment response was seen in significantly more participants in the intervention (4/20; 20%) compared to the control group (0/20, 0%; *χ*^2^ (2)(*N* = 40) = 4.44; *p* < 0.02). At the 6-month follow-up, effects were maintained for depressive symptom reduction (*d*_pr6f_ = 0.71; 95% CI: 0.19-1.61) but not treatment response.

**Conclusion:**

Disease-specific severity indicators were not related to a differential effectiveness of guided self-help for depression and diabetes. Clinical meaningful effects were observed in nonsuicidal severely depressed individuals, who do not need to be excluded from web-based guided self-help. However, participants should be closely monitored and referred to other treatment modalities in case of nonresponse.

## 1. Introduction

Prevalence of diabetes mellitus in the general population is high. In 2014, according to the World Health Organization, 422 million adults worldwide (8.5%) were suffering from diabetes [1]. Individuals with diabetes mellitus are highly burdened and often have increased health problems, resulting in high socioeconomic costs and a higher frequency of medical and psychological comorbidities. A common comorbidity is major depressive disorder (MDD), which affects 10 to 20% of adult diabetes patients, resulting in poorer diabetes self-management, poorer general health outcome, higher frequency of secondary diseases, decreased quality of life, and a higher mortality rate.

Treatment options are available for MDD and diabetes separately, but only few specialized treatments exist that take both diabetes and depression into account. The majority of “double troubled” individuals remain untreated for depression, calling for integration of depression treatment in routine diabetes care [2]. Internet- and mobile-based interventions (IMIs) may be helpful in this context offering advantages over traditional psychological services. IMIs provide the following: (1) easy accessibility at any time and place; (2) possible anonymity if desired; (3) individuals can work at their own pace and have the opportunity to review materials as often as they want; (4) easy scalability; thus, only a small increase of resources is required for reaching a greater proportion of the eligible population using these interventions; and (5) reaching populations that may not partake in existing traditional onsite interventions [3].

In the last decade, a large number of studies have documented the effectiveness of IMIs in mental disorders and specifically MDD [4–6]. To the best of our knowledge, two existing web-based interventions for comorbid depression in diabetes have been tested: Diabetergestemd.nl (DbG.nl) [7] and Get.On Mood Enhancer Diabetes (GET.ON M.E.D.) [8]. Both interventions consisted of a minimally guided self-help programme. GET.ON M.E.D. additionally used a standardized text message-based coach to foster adherence and facilitate the transfer of training elements in daily life routine. Diabetergestemd has shown to be effective in reducing depressive symptoms after treatment and after a 1-month follow-up (*d*_fu_ = 0.29; 95% CI: 0.17-0.40) compared to a waitlist control group as well as a greater reduction in diabetes-related emotional distress. The intervention had no beneficial effect on glycemic control. Similar evidence was provided by Nobis et al. [9], in which GET.ON M.E.D. was effective in reducing depressive symptoms (*d* = 0.89; 95% CI: 0.64-1.15) and diabetes-related emotional distress (*d* = 0.58; 95% CI: 0.33-0.83) at posttreatment when compared to an online psychoeducation intervention. Effects of improvements in depressive symptom severity were sustained at the 6-month follow-up (*d* = 0.83; 95% CI: 0.6-1.1) [10].

Despite the evidence for effective IMIs for depression [4] and evolving evidence for the effectiveness of IMIs for comorbid depression in diabetes [7, 9], it remains unclear, which groups of participants profit from such interventions [11] and who should be offered other treatment delivery formats. The exploration of effect modifiers of the treatment outcome can help identify groups of patients who do and those who do not profit. However, research on effect modifiers is scarce. Interestingly, a recent individual participant data meta-analysis found that participants with severe depressive symptoms at the baseline were more likely to remit after participation in Internet-based treatment than those with milder symptomatology (Odds Ratio (OR) = 1.19; 95% CI: 1.01-1.39) [5]. Furthermore, older participants (OR = 1.01; 95% CI: 1.00-1.02) and native-born participants (OR = 1.66; 95% CI: 1.07-2.59) were more likely to respond to treatment compared to younger participants and ethnic minorities, respectively. Age (OR = 1.01; 95% CI: 1.00-1.02) and ethnicity (OR = 1.73; 95% CI: 1.07-2.81) also moderated the effects of treatment on remission. The results from this comprehensive study do not suggest that participants with severe depression should be excluded from IMIs, at least not from an efficacy point of view. Of course, safety is an issue that deserves to be determined, but harmful effects have not been documented. Little is known about effect modification in individuals with depression and comorbid somatic diseases, and it is important to investigate whether depression and disease-specific severity indicators are associated with differential treatment outcomes of Internet- and mobile-based interventions.

Knowing whether more severely depressed individuals with diabetes may profit or not from an IMI is key to provide adequate, tailored treatment options with low risk of harm and high chance of treatment success. To evaluate treatment modifiers, van Bastelaar et al. [12] compared subgroups of individuals with diagnosed diabetes (mix of type 1 and type 2) with and without clinically relevant depression, anxiety disorder, and diabetes-specific emotional distress in secondary analyses of a RCT. No different effects of the intervention between subgroups of participants were found, indicating that participants with a more clinical profile profited to a similar extent. To explore whether the findings of van Bastelaar et al. [12] are applicable for our GET.ON M.E.D. study population, we investigated disease-specific severity indicators as the treatment outcome modifier.

In the present study, we aim to evaluate the effectiveness of a minimally guided web-based self-help intervention for comorbid depression in individuals with diabetes with regard to treatment predictors and moderators and specifically with regard to participants with nonsuicidal severe depression. The primary research questions were the following: (1) Do disease-specific severity indicators (i.e., MDD diagnosis baseline, depressive symptom severity, glucose level, and diabetes-related emotional distress) predict and/or moderate the effectiveness of GET.ON M.E.D. compared to an online psychoeducation control group (CG)? (2) What are the specific effects of GET.ON M.E.D. in nonsuicidal severely depressed individuals when compared to online psychoeducation?

## 2. Methods and Materials

### 2.1. Study Design

All secondary analyses were conducted with data from a randomized controlled trial (*N* = 253) examining the effectiveness of a minimally guided self-help intervention for individuals with diabetes mellitus type 1 or type 2 and comorbid depression compared to an online psychoeducation control condition [7–9]. To examine for which severity level the IMI can be recommended, moderation analysis and subsequent subgroup analysis were conducted. Study procedure for the primary study was approved by the ethical board of the University of Marburg (2012-45K) and registered in the German registry of clinical trials (DRKS00004748). All study outcomes except for the structured clinical interview for DSM-IV (SCID) [13] were measured using self-report assessments at the baseline (T1), posttreatment (T2), and a 6-month follow-up (T3). All self-report assessments used a secure online-based assessment system (AES, 256-bit encrypted).

### 2.2. Participants

Patients were eligible for the study if they (a) were at least 18 years old, (b) met criteria for diabetes mellitus type 1 or type 2 ICD-10 [14], (c) had moderate or high depressive symptoms (Center for Epidemiological Studies Depression Scale (CES − D ≥ 23) [15, 16]), (d) had sufficient German language proficiency, and (e) had access to a computer with Internet and a valid email address as well as a phone able to receive text messages. Exclusion criteria included (a) a notable suicidal risk indicated by a score greater than 1 on the Beck Depression Inventory II (BDI-II) item no. 9 [17, 18] and (b) current psychotherapeutic treatment or being on a waiting list for such treatment. All participants had unrestricted access to treatment as usual in their mental health and routine diabetes care. Study recruitment, enrollment, and use of other mental healthcare are documented in Nobis et al. [8, 9].

#### 2.2.1. Intervention

Participants in the intervention group (IG) had access to the online intervention GET.ON M.E.D. (for a detailed description, see Nobis et al. [8]). The online intervention consisted of six minimally guided sessions with approximately one session à 45 to 60 minutes per week. There are two optional sessions on weight and sleep problems as well as one booster session, four weeks after completion of the intervention. GET.ON M.E.D. is evidence-based on two key elements: systematic behavioral activation [19] and problem solving [20]. Each session includes diabetes-specific content in relation to depression, such as worries about disease complications, physical activity, blood glucose, patient-physician relation, and sexuality [21, 22]. Each participant was guided by a trained psychologist (eCoach) and received feedback on the online sessions as well as reminders in case of not completing the session. Participants had the option to receive a set of daily standardized text messages to support transferring learned skills into their daily routine.

#### 2.2.2. Psychoeducation Control Group

Participants in the control group (CG) had access to an online psychoeducation based on the guidelines for MDD of the German Physicians Association [8, 23]. Psychoeducation included basic information about depression, symptoms, and possible ways to find treatment or help. After study completion (twelve months after randomization), participants of the control group received unguided access to GET.ON M.E.D.

### 2.3. Outcomes

#### 2.3.1. Primary Outcome: Depressive Symptoms (CES-D)

The primary outcome of the primary study was depressive symptom severity measured with the German version of the Center for Epidemiological Studies-Depression (CES-D) [15]. The items refer to the previous week and are answered on a 4-point Likert-type scale, from 0 (rarely or none of the time) to 3 (most or all of the time). Total scale scores range from 0 to 60. Scores of CES − D ≥ 16 indicate relevant levels of depression severity, and a score of ≥23 indicates the clinical level of depression severity. In this study, Cronbach's alpha was 0.80. To determine severity cutoffs, we used the conversion scores from the widely used and extensively evaluated cutoff scores of the Beck Depression Inventory II [17, 18] according to the formula 1.13 + (0.68) × (CI = ±13.1) by González and Jenkins [24]. The conversion was needed to correctly identify participants with self-reported severe levels of depression as the CES-D does not differentiate for different clinical severity levels. After conversion, the following cutoffs for depression severity for the German CES-D were determined: no depression (≤16) and severe depression (≥40) [15].

#### 2.3.2. Moderators

In addition to depressive symptom severity, the following moderators were assessed:

- *MDD at the Baseline* (*SCID*). To assess the diagnostic status of the participants, the structured clinical interview for DSM-IV (SCID) was conducted at the baseline [13, 25]. Trained psychologists administered the interview over the phone.
- *Most Recent Blood Glucose Level* (*HbA_1c_*). HbA_1c_ as the indicator of overall diabetes control was assessed at the baseline by self-report and referred back to the previous six to eight weeks. Healthy people have an HbA_1c_ under 39 mmol/mol or 5.7%. Values from 48 mmol/mol (6.5%) and up indicate diabetes. The German Diabetes Society recommends to aim for HbA_1c_ under 58 mmol/mol (7.5%) for type 1 [26] and between 48 and 58 mmol/mol (6.7-7.5%) for type 2 diabetes [27].
- *Diabetes-Related Emotional Distress*. Current emotional distress related to living with diabetes was assessed by the Problem Areas in Diabetes [28] scale short form [29]. It consists of 5 items and is rated on a 5-point Likert scale ranging from “no problem” to “serious.” A score higher than 7 shows elevated diabetes-related emotional distress [29]. The validity and reliability were shown to be good (*α* = 0.83). In this study, Cronbach's alpha was 0.84.

### 2.4. Statistical Analysis

Prediction, multiple moderation, and nonsuicidal severely depressed subgroup analyses were conducted. All analyses were performed with IBM Statistics 24. Missing data were imputed using a Markov chain Monte Carlo [30] multivariate imputation algorithm with 10 estimations per missing value in accordance with the intention-to-treat principle (ITT). All analyses were conducted in accordance with the Consolidated Standards of Reporting Trials (CONSORT) statement [31].

#### 2.4.1. Predictor and Multiple Moderation Analyses

To determine the effects of disease-specific severity indicators (i.e., depressive symptom severity, diabetes-related emotional distress, and HbA_1c_) on the effectiveness of the intervention, multiple moderation analysis (MMA) with the SPSS macro PROCESS by Hayes [32] was conducted. By using the difference in change of the outcome to postassessment and 6-month follow-up as a dependent variable, it was controlled for baseline differences in the prediction, moderation, and clinical subgroup analyses. Based on separate multiple regressions, MMAs test three elements to predict the outcome: the baseline variable's main effect, the treatment condition effect (IG, CG), and the interaction effect of the baseline variable and treatment condition. Analyses yielding a significant main effect of only the baseline variable indicated that the baseline variable was a predictor with regard to difference in change between the study conditions [33], for which the direction of the effect will be presented. If the outcome is significantly predicted by the interaction effect, it can be regarded as a moderator. Effect modifiers (moderators) indicate that populations benefit significantly different from each other depending on the investigated severity indicator. Moderators and predictors of the difference in change of the outcomes were presented for posttreatment and follow-up. If significant moderators were identified, one of two subsequent analyses was performed: (1) for dichotomous variables, subsequent simple slope analyses were conducted. Simple slope analyses were run to explore the direction of an interaction effect, improve interpretability, and provide specific estimations for the investigated subgroups of interest. For this, the data was split into subgroups based on conditional values (i.e., yes and no), and outcomes in each subgroup were explored. (2) In case of continuous variables, the region of significance (AOS) via the Johnson-Neyman technique was calculated to explore significant transition points within the moderator. For prediction and moderation analyses, hypotheses were bidirectional and were therefore tested two-sided.

#### 2.4.2. Nonsuicidal Severely Depressed Subgroup Analysis

Inclusion was defined by a cutoff of CES − D ≥ 40 (through converted BDI-II cutoff of 29 for severe depression), which refers to severe depression. An analysis of variance with simple effects was used to compare outcomes between groups at posttreatment and at 6-month follow-up. Results were reported as mean within- and between-group differences and as Cohen's *d* effect sizes [34] (and their 95% CIs, according to Hedge and Olkin [35]). Interpretation of Cohen's *d* is as follows: small effects (*d* = 0.2), medium effects (*d* = 0.5), and large effects (*d* = 0.8) [34]. Improvements on the primary outcome at the individual level were examined by assessing the number of participants who displayed treatment response and near-to-symptom-free status. Treatment response was defined by the reliable change index (RCI) as proposed by Jacobson and Truax [36] and as at least 50% reduction in the symptom score from the baseline to postassessment and 6-month follow-up, respectively, on the CES-D. According to Jacobson and Truax [36], the reliable change was calculated by using the following formula: 1.96 × SD1 × sqrt(2) × sqrt (1-rel). Participants with a reliable positive change in depression (RCI > 1.96, ≥8.99 CES-D points) were classified as responders. Accordingly, symptom deterioration was classified as increase from posttreatment to 6-month follow-up by 8.99 CES-D points. In addition, we examined how many participants reached a near-to-symptom-free status at postassessment and 6-month follow-up as indicated by a CES-D score ≤ 16. The number needed to treat (NNT) [37] with GET.ON M.E.D. to achieve one treatment response or near-to-symptom-free status, respectively, as compared to the control group was also calculated to estimate the clinical effect size. Hypotheses were one-directional and were thus tested one-sided. Differences between the intervention condition and the control group were tested in a chi-square (*χ*^2^) test.

## 3. Results

### 3.1. Descriptive Statistics

In total, 253 participants were included in the secondary analyses in comparison to 256 in the original effectiveness analysis, as data from three participants was not eligible for secondary analyses, due to missing baseline assessments. Participants were predominately females (*n* = 159; 62.8%) with an average age of 50.7 (SD = 11.7). The majority of participants (62.8%, *n* = 159) had a midlevel education and were Caucasians (74.3%, *n* = 188). A total of 44.6% (*n* = 113) participants were reported to be diagnosed with diabetes mellitus type 1, and the other 55.3% (*n* = 140) were diagnosed with diabetes mellitus type 2. 43.8% (*n* = 111) took medication for diabetes; in 21.3% (*n* = 55), the medication was insulin. The average time since their diabetes was diagnosed was between 5 and 10 years (range: 0.25 years–over 10 years). A total of 24.9% (*n* = 63) were diagnosed with diabetes-related complications, such as high blood pressure and nerve damage (for further information on total sample and subgroup characteristics, see Table 1). The mean scores for outcomes are reported in Table 2.

### 3.2. Predictor and Multiple Moderation Analyses

Baseline MDD did not predict improvement in depressive symptom severity from the baseline to posttreatment (*β* = 1.72, *p* = 0.08); however, improvement was predicted from the baseline to follow-up (*β* = 2.54, *p* = 0.01). Furthermore, higher levels of depressive symptom severity at the baseline were associated with greater reduction in depressive symptom severity to posttreatment (*β* = 0.50, *p* = 0.00) and follow-up (*β* = 0.45, *p* = 0.00). Lower levels of HbA_1c_ were associated with greater improvement in depressive symptom severity from the baseline to posttreatment (*β* = −0.77, *p* = 0.04) but not from the baseline to follow-up (*β* = −0.41, *p* = 0.27). Diabetes-related emotional distress did predict the treatment outcome neither at posttreatment (*β* = 0.02, *p* = 0.83) nor at follow-up (*β* = −0.04, *p* = 0.68). For further information, see Table 3.

None of the examined variables significantly moderated the treatment outcome, neither at posttreatment nor at follow-up (*p* ranging from 0.41 to 0.99; for detailed information, see Table 3). Simple slope analysis and region of significance are not reported due to nonsignificance.

### 3.3. Nonsuicidal Severely Depressed Subgroup Analysis

In total, 40 participants (IG = 20; CG = 20) fulfilled criteria for severe depression (CES − D ≥ 40, *M* = 43.45, range: 40-53, SD = 3.17) and were investigated in the nonsuicidal severely depressed subgroup analysis. It revealed that nonsuicidal severely depressed participants in the intervention group benefitted substantially from the intervention in terms of reduction of depressive symptom severity (*F*(2, 76) = 4.47, *p* = 0.017, *d* = 0.67, CI: 0.03-1.30) when compared to the control group. In the intervention group, simple effect analysis showed a statistically significant reduction in depressive symptom severity from the baseline to posttreatment (*p* < 0.001, *d* = 2.17, CI: 1.39-2.96) and to 6-month follow-up (*p* < 0.001, *d* = 1.60, CI: 0.93-2.37), which was greater than in the control group (baseline to posttreatment: *p* = 0.001, *d* = 0.92, CI: 0.001-1.83; baseline to 6-month follow-up: *p* = 0.001, *d* = 1.17, CI: 0.22-2.12). Between-group effect size was *d* = 1.05 (CI: 0.11-1.98) at posttreatment and *d* = 0.71 (CI: 0.19-1.61) at 6-month follow-up.

The reliable change index at posttreatment showed that in the IG, 15 participants (75%) improved at posttreatment. Achieved changes were maintained at the 6-month follow-up with twelve (60%) participants maintaining achieved changes from posttreatment and two (10%) participants who improved from posttreatment to follow-up. At posttreatment in the CG, eight participants (40%) improved, which was maintained at the 6-month follow-up by seven (35%) participants. Two (10%) participants improved from posttreatment to follow-up. The difference between the groups at posttreatment was statistically significant (*χ*^2^ (1) = 5.013; *p* = 0.012). The difference between the groups at the follow-up was not statistically significant (*χ*^2^ (1) = 2.50; *p* = 0.056, ns.). To achieve one additional treatment response as compared to the control group, 2.9 participants have to be treated with this intervention.

Positive treatment response in terms of reduction of depressive symptom severity (CES-D reduction of 50%) at posttreatment was higher in the intervention group with four participants (20%), whereas no participant (0%) in the CG showed a positive treatment response. This difference was statistically significant (*χ*^2^ (2) = 4.44; *p* = 0.017). From posttreatment to follow-up, one (5%) participant of each group showed a positive treatment response (ns.). To achieve one additional positive treatment response, five participants have to be treated with this intervention.

Near-to-symptom-free status was not achieved in either groups at posttreatment; two (10%) participants in the IG achieved near-to-symptom-free status at the 6-month follow-up as compared to none (0%) in the CG. Near-to-symptom-free status was not statistically significantly different between the groups at follow-up assessment (*χ*^2^ (1) = 2.10; *p* = 0.07, ns.). To achieve one additional near-to-symptom-free status at follow-up treatment, ten participants have to be treated.

Symptom deterioration did not take place in the intervention group at posttreatment (0%). In the CG, one participant (5%) did experience deterioration. This difference was not statistically significant (*χ*^2^ (1) = 1.02; *p* = 0.15, ns.). At the follow-up, three participants (15%) in the IG experienced a deterioration as compared to one (5%) in the control group; this difference was not statistically significant (*χ*^2^ (1) = 1.11; *p* = 0.14, ns.).

## 4. Discussion

This study is aimed at exploring whether disease-specific severity indicators predict and moderate treatment outcome in a 6-week minimally guided web-based self-help intervention on depression and diabetes and its specific effectiveness in participants with severe symptoms of nonsuicidal depression.

### 4.1. Main Findings

MDD at the baseline, depression severity, and average blood glucose control significantly predicted the treatment outcome. Although multiple moderation analysis did not reveal any significant moderators, nonsuicidal severely depressed subgroup analysis showed that the most burdened participants profited substantially from the intervention with medium to large between-group effects on reduction in depressive symptoms compared to the control group at posttreatment and at 6-month follow-up. With regard to the diabetes control, as indicated by HbA_1c_, we found HbA_1c_ to be a significant predictor but not a moderator.

### 4.2. Comparison to Previous Research

Our findings on predictors and moderators of the treatment outcome are in line with other studies. The individual participant data meta-analysis by Karyotaki et al. found that participants with severe depressive symptoms at the baseline were more likely to remit, which also means that participants with severe depressive symptoms do profit from Internet-based treatment [5]. We found similar results in participants with severe depression and a somatic comorbidity. We did not find any modifying effects of baseline severity indicators in this sample, which endorses the results of van Bastelaar et al. [12]. GET.ON M.E.D. thus does not seem to be especially more or less helpful to one particular subgroup of participants. The higher symptom severity is not necessarily associated with the worse treatment outcome in a guided self-help intervention [38, 39]. This study extends previous findings, that high disease burden in individuals can be managed within this type of intervention [40] to individuals with somatic comorbidities.

Our findings on the effectiveness of GET.ON M.E.D. in the nonsuicidal severely depressed subgroup are supported by studies which show that Internet-based self-help interventions can be effective in clinical populations [4, 41]. Results from subgroup analyses also revealed that nonsuicidal severely depressed participants in the intervention group were significantly more likely to have a greater reduction in depressive symptom severity than participants of the control group (*d*_post_ = 1.05, *d*_6fu_ = 0.71). This is shown by the NNT of 2.9 in the clinical subgroup which is comparable to the NNT of 2.2 in the effectiveness trial [9]. The comparable NNTs are in line with findings from van Bastelaar et al. [12], that participants with subclinical and clinical symptoms may profit the same from minimally guided online interventions.

To the best of our knowledge, previous studies did not include the HbA_1c_ in multiple moderation analyses. We found HbA_1c_ to be a significant predictor but not a moderator. It should however be taken into account that most participants' values for the HbA_1c_ were already in the normal range at the baseline, so that baseline variance in HbA_1c_ values may be too low to detect potential effects on changes in depression.

### 4.3. Limitations

The following limitations have to be considered: First, participants with a potential risk of acute suicidal ideation were excluded, thereby limiting the findings to a nonsuicidal severely depressed subgroup. This limits the generalizability of our results as severe depression is often accompanied by risk of acute suicidal ideation. There is no scientific evidence on specific exclusion criteria for participants within online interventions, although exclusion of potential participants with specific conditions may be reasonable (e.g., acute suicide risk and psychotic episodes). It remains unclear, which exclusion criteria should be considered with regard to a specific population. Second, participants were recruited from the general population, meaning generalizability to other settings in which patients seek help in routine clinical mental health care might be limited. Furthermore, this study was not a priori planned to detect potential moderators [42–44] and to conduct subgroup analysis in nonsuicidal severely depressed participants. Studies show that one needs approximately three times as many participants to detect moderators with a similar effect size in a three-way interaction compared to simple two-way interaction [45]. However, the initial study was planned to detect effects of 0.35; therefore, 260 individuals were included. Yet, the observed effect size in the initial study was *d* = 0.89 for differences in depression severity at posttreatment. Therefore, the sample size allowed us to detect moderators with a medium to large effect but may be still too small to detect moderators with only small effects. Third, no clinical interviews took place at posttreatment or 6-month follow-up. Therefore, changes in the diagnosis of MDD could not be analyzed. Fourth, the data used in this trial was collected in a previous trial, so that only predictors and moderators which were included in the original design could be evaluated.

### 4.4. Implications for Clinical Practice and Future Research

The results from this study support evidence that nonsuicidal severely depressed individuals can profit from low-threshold interventions and extend the findings to nonsuicidal severely depressed individuals with a somatic comorbidity. In our sample, 6.5% of all participants were excluded from the study due to an elevated level of suicidal ideation, increasing the likelihood of suicidal behavior. Seemingly, individuals interested in IMIs may have a lower association between severe depression and suicidal ideation compared to individuals that prefer traditional treatment options. Safety management may therefore be a valid option, if the IMI may be implemented in routine care. However, future research should include severely depressed individuals with suicidal ideation to examine which safety management should be used for suicidal behavior prevention within IMIs.

For severe depression, current treatment guidelines [23, 46] recommend a combination of pharmacological and psychotherapeutic treatments. Effects of pharmacological antidepressant treatment are typically seen after two to four weeks [47]. Treatment continuation is recommended for four to nine months [23]. Despite the well-proven effectiveness of pharmacological antidepressant treatment [48, 49], side effects can be considerably high. Depending on the type of antidepressant, side effects can occur within the cardiovascular system (e.g., heart rate and heart rate variability) and the gastrointestinal system and can cause agitation as well as sexual dysfunction [50–53]. Side effects are especially disadvantageous for individuals with diabetes due to the high impact diabetes can have on the human body. Antidepressant medication may worsen preexisting somatic problems such as high blood pressure and coronary heart disease in individuals with diabetes. Therefore, IMIs might be beneficial for individuals with severe somatic comorbidities. However, some existing treatment guidelines for depression only recommend low-intensity psychological interventions such as IMIs only to manage (persistent) depressive symptoms and mild to moderate depression [23, 46]. Findings from our study showed, however, that IMIs could be considered as a viable treatment option for a wide group of patients with diabetes, and even patients with nonsuicidal moderate to severe depression do not need to be excluded. Nevertheless, future research should focus on researching exclusion criteria for low-threshold IMIs if safety management is not an option.

Although IMIs do not cause side effects on a somatic level, participation might be accompanied by negative effects. Such negative effects might include negative changes in relationships with friends and family and negative changes in effect due to the IMI itself [54, 55]. However, research on negative effects in IMIs is still scarce. Yet, negative effects do occur not only in IMIs but also in traditional face-to-face psychotherapy.

From a health economic perspective, there are no studies available that show the cost-effectiveness of antidepressant medication and IMIs. There are several studies that show the cost-effectiveness of an antidepressant medication [56]. There is also emerging evidence that IMIs for depression can be cost-effective, although findings are still inconsistent [57]. Yet, a health economic evaluation showed that GET.ON M.E.D. demonstrated a high probability of being cost-effective compared to enhanced usual care [58].

Since a direct comparison of pharmacological treatment and IMIs in terms of clinical and cost-effectiveness is not yet available, it is important to investigate which kind of treatment is beneficial for which group of patients. IMIs for patients with diabetes should be considered, when pharmacological antidepressant treatment is not an option. However, not every severely depressed participant profits from an IMI, so further research is needed to better identify severely depressed participants that are likely to profit from low-threshold interventions such as IMIs and to identify nonresponders to be able to refer them to other treatment formats.

### 4.5. Conclusion

In sum, it can be concluded that a minimally guided online self-help intervention is effective in reducing depressive symptom severity in participants with lower as well as higher levels of depressive symptom severity. This indicates that nonsuicidal severely depressed participants with somatic comorbidities do not need to be excluded from online interventions as partaking in such interventions can be beneficial, especially within stepped care approaches.

## Figures and Tables

**Table 1:** Demographics.

Variable	All participants			Nonsuicidal severely depressed subgroup		
	IG	CG	Total	IG	CG	Total
	(*N* = 127)	(*N* = 126)	(*N* = 253)	(*N* = 20)	(*N* = 20)	(*N* = 40)
Age: mean (SD)	50.16 (11.68)	51.34 (11.92)	50.75 (11.7)	44.45 (11.24)	47.20 (13.37)	45.83 (12.35)
Sex: female (%)	80 (63.0)	79 (62.7)	159 (62.8)	13 (65.0)	14 (70.0)	27 (67.5)
Education (%)						
High	32 (25.2)	45 (35.7)	30.4 (77.0)	2 (10.0)	6 (30.0)	8 (20.0)
Medium	87 (69.0)	72 (57.1)	159 (62.8)	16 (80.0	11 (55.0)	27 (67.5)
Low	7 (5.5)	9 (7.1)	16 (6.3)	2 (10.0)	3 (15.0)	5 (12.5)
Married (%)	84 (66.1)	75 (59.5)	159 (62.8)	10 (50.0)	8 (40.0)	18 (45.0)
Ethnicity (%), Caucasian	94 (74.0)	94 (74.6)	188 (74.3)	15 (75.0)	16 (80.0)	31 (77.5)
Diabetes (%)						
Diabetes I	64 (50.4)	49 (38.9)	113 (44.6)	14 (70.0)	8 (40.0)	22 (55.0)
Diabetes II	63 (49.6)	77 (61.1)	140 (55.3)	6 (30.0)	12 (60.0)	18 (45.0)
Medication, yes (%)	50 (39.3)	61 (48.4)	111 (43.8)	5 (25.0)	10 (50.0)	15 (37.5)
Insulin, yes (%)	29 (58.0)	26 (42.6)	55 (49.5)	3 (60.0)	6 (60.0)	9 (60.0)
Average years since diabetes diagnosis	5-10 years	5-10 years	5-10 years	>10 years	5-10 years	>10 years
Secondary diseases (%)	33 (26.0)	30 (28.3)	63 (24.9)	6 (30.0)	5 (25.0)	11 (27.5)
High blood pressure	15 (45.4)	12 (40.0)	27 (42.8)	3 (50.0)	2 (40.0)	5 (45.4)
Coronary heart disease	4 (12.1)	4 (13.3)	8 (12.6)	—	—	—
Blood vessels	9 (27.2)	8 (26.6)	17 (26.9)	1 (16.6)	1 (20.0)	2 (18.1)
Eye disease	13 (39.3)	5 (16.6)	18 (28.5)	3 (50.0)	3 (60.0)	6 (54.5)
Nerve damage	13 (39.3)	11 (36.6)	24 (38.0)	4 (66.6)	3 (60.0)	7 (63.6)
Kidney damage	3 (9.0)	2 (6.6)	5 (7.9)	—	—	—
Infections	5 (15.1)	3 (10.0)	8 (12.6)	1 (16.6)	—	1 (9.0)
Sexual dysfunction	9 (27.2)	8 (26.6)	17 (26.9)	3 (50.0)	1 (20.0)	4 (36.3)
Eating disorder	5 (15.1)	6 (20.0)	11 (17.4)	1 (16.6)	3 (60.0)	4 (36.3)
Other	7 (21.2)	4 (13.3)	11 (17.4)	1 (16.6)	1 (20.0)	2 (18.2)
Experience with psychotherapy (%)	69 (54.8)	71 (56.3)	140 (55.3)	9 (45.0)	13 (65.0)	22 (55.0)
Psychotherapy for depression	48 (69.5)	52 (73.2)	100 (71.4)	7 (77.7)	8 (61.5)	15 (68.1)

IG: intervention group; CG: control group. Percentages less than 100 are due to missing data.

**(a):** 

All participants									
Variable	IG (*N* = 127)			CG (*N* = 126)			Total (*N* = 253)		
	T1	T2	T3	T1	T2	T3	T1	T2	T3
CES-D (SD)	32.06 (6.76)	21.05 (8.90)	19.58 (9.38)	31.54 (7.53)	28.85 (8.75)	26.76 (9.56)	31.80 (7.14)	24.94 (9.62)	23.14 (10.11)
MDD diagnosis at baseline (%)									
Yes	73 (57.4)	—	—	66 (52.3)	—	—	139 (54.9)	—	—
No	54 (42.5)	—	—	60 (47.6)	—	—	114 (45.1)	—	—
Converted: BDI-II (SD)	22.93 (4.59)	15.44 (6.05)	14.44 (6.38)	22.58 (5.12)	20.75 (5.93)	19.31 (6.50)	22.75 (4.86)	18.09 (6.54)	16.87 (6.80)
Average blood glucose level, HBA_1c_ (SD)	7.55 (1.62)	—	7.42 (1.59)	7.35 (1.30)	—	7.40 (1.32)	7.45 (1.47)	—	7.41 (1.44)
PAID (SD)	10.24 (4.28)	8.27 (3.76)	7.94 (4.26)	10.60 (4.53)	10.91 (4.66)	9.80 (4.52)	10.42 (4.40)	9.59 (4.42)	8.86 (4.48)

**(b):** 

Nonsuicidal severely depressed subgroup									
Variable	IG (*N* = 20)			CG (*N* = 20)			Total (*N* = 40)		
	T1	T2	T3	T1	T2	T3	T1	T2	T3
CES-D (SD)	42.85 (2.99)	29.33 (8.25)	28.64 (11.79)	44.05 (3.31)	38.12 (8.48)	36.07 (8.99)	43.45 (3.17)	33.72 (9.38)	32.35 (11.01)
MDD diagnosis at baseline (%)									
Yes	15 (75.0)	—	—	17 (85.0)	—	—	32 (80.0)	—	—
No	5 (15.0)	—	—	3 (15.0)	—	—	8 (20.0)	—	—
Converted: BDI-II (SD)	30.26 (2.03)	21.07 (5.61)	20.60 (8.02)	31.08 (2.25)	27.05 (5.76)	25.65 (6.11)	30.67 (2.16)	24.06 (6.37)	23.13 (7.49)
Average blood glucose level, HbA_1c_ (SD)	7.63 (1.89)	—	8.52 (1.84)	7.72 (1.63)	—	7.63 (1.64)	7.67 (1.74)	—	7.97 (1.73)
PAID (SD)	11.05 (5.30)	9.80 (3.96)	10.01 (4.96)	13.70 (4.85)	14.17 (4.58)	13.23 (4.50)	10.42 (4.40)	9.59 (4.42)	8.86 (4.48)

CES-D: Center for Epidemiological Studies-Depression; MDD: major depressive disorder; BDI-II: Beck Depression Inventory II, PAID: Problem Areas in Diabetes.

**(a):** 

Change in depressive symptom severity (CES-D)												
Potential predictors	Pre post						Pre 6fu					
	*b*	se	*t*	*p*	LLCI	ULCI	*b*	se	*t*	*p*	LLCI	ULCI
Depression diagnosis: MDD	1.72	0.99	1.73	0.08	-0.23	3.36	2.54	1.05	2.40	**0.01** ^∗^	0.46	4.46
Depressive symptom severity: con. BDI-II	0.50	0.10	4.92	**0.00** ^∗∗^	0.30	0.70	0.45	0.11	3.92	**0.00** ^∗∗^	0.22	0.69
Glucose level: HbA_1c_	-0.77	0.38	-2.02	**0.04** ^∗^	-1.53	-0.02	-0.41	0.37	-1.10	0.27	-1.15	9.65
Diabetes related distress: PAID	0.02	0.11	0.20	0.83	-0.20	0.25	-0.04	0.11	-0.40	0.68	-0.28	0.18

**(b):** 

Change in depressive symptom severity (CES-D)												
Interaction with group and potential moderator	Pre post						Pre 6fu					
	*b*	se	*t*	*p*	LLCI	ULCI	*b*	se	*t*	*p*	LLCI	ULCI
Depression diagnosis: MDD	1.60	1.98	0.82	0.41	-2.26	5.53	1.49	2.11	0.70	0.48	-2.67	5.66
Depressive symptom severity: con. BDI-II	-0.01	0.20	-0.08	0.93	-0.41	0.38	-0.05	0.23	-0.24	0.80	-0.51	0.40
Glucose level: HbA_1c_	0.14	0.76	0.18	0.85	-1.36	1.65	0.09	0.75	0.12	0.90	-1.38	1.57
Diabetes related distress: PAID	0.00	0.23	0.00	0.99	-0.45	0.46	-0.07	0.23	-0.31	0.75	-0.54	0.39

CES-D: Center for Epidemiological Studies-Depression; MDD: major depressive disorder; con.: converted; BDI-II: Beck Depression Inventory II; PAID: Problem Areas in Diabetes. ^∗^*p* < 0.05; ^∗∗^*p* < 0.01.

## Data Availability

Previously reported data were used to support this study (see 10.2337/dc14-1728, 10.1111/dme.13173 and 10.1186/1471-244X-13-306). These prior studies are cited at relevant places within the text as references [8–10].
